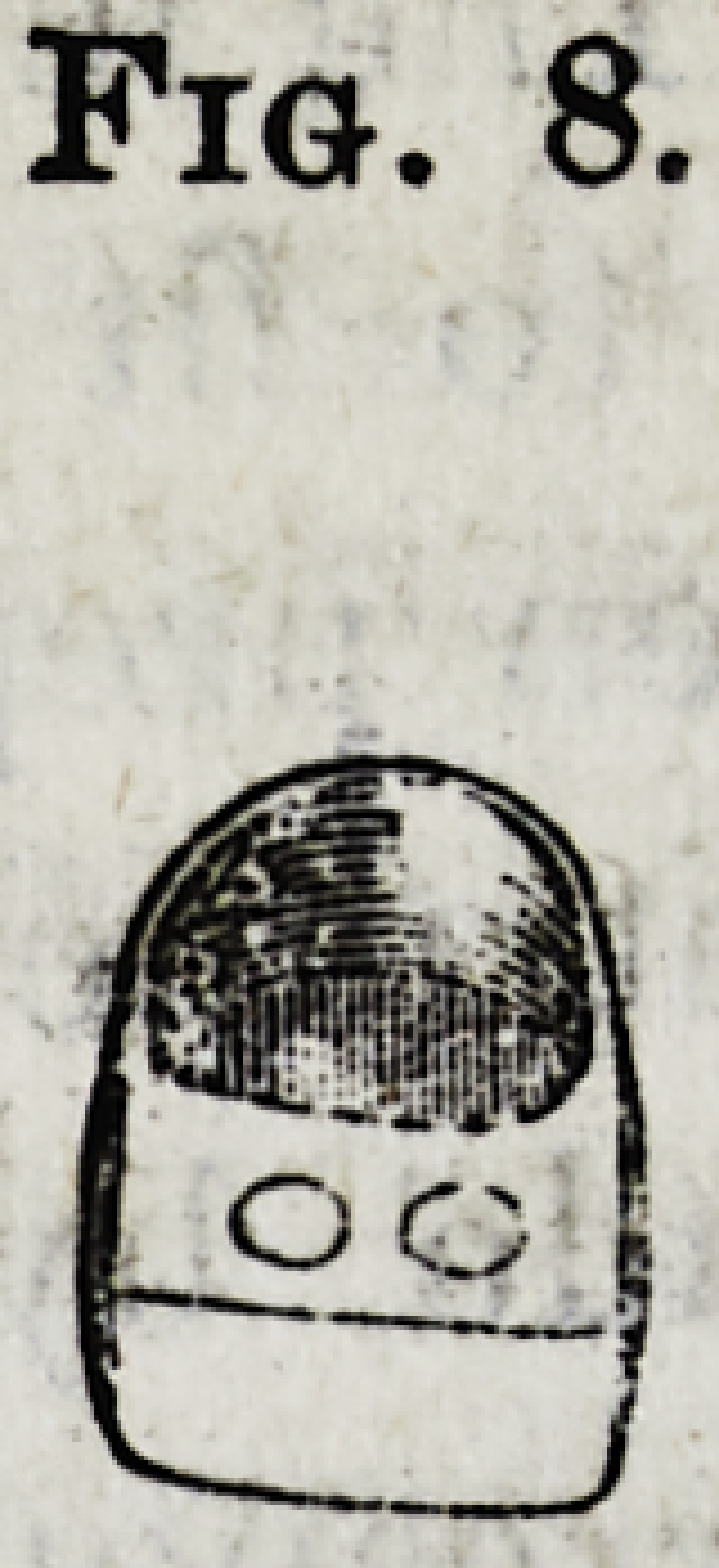# Attachment of Artificial Crowns to Natural Roots

**Published:** 1883-03

**Authors:** H. W. F. Buttner

**Affiliations:** New York, N. Y.


AMERICAN JOURNAL

OF

DENTAL SCIENCE.

Vol. XVI. THIRD SERIES—MARCH, 1883. No. 11

### ARTICLE I.

### *Attachment of Artifical Crowns to Natural Roots.*

BY H. W. F. BUTTNER, D. D. S., NEW YORK, N. Y.

[Read before the New York Odontological Society, October 17, 1882.]

Although this subject had been extensively discussed, yet many will agree with me that the problem had not been satisfactorily solved. The great value of pivoting operations has long been recognized, and has been one of the most important questions for the last thirty years. In looking over the dental journals we find a great many varieties of attachments for artificial crowns upon natural roots have been described. In order to get a clear idea of the importance of this problem, and to give due credit to those who have contributed to the general knowledge, I will, before entering upon the description of my method, give a brief sketch of a variety of processes, as published in the journals. Although there is a distinctive difference in these processes, they may be divided into two general classes.

The first, and doubtless the oldest method, is that which depends upon a central pivot of wood or metal, to hold the artificial crown in position.

The second class consists of methods in which, besides a central pivot a ring is fitted around the root, to increase durability and prevent fracture.

HISTORY.

The first who introduced the latter method to the profession was Dr. W. H. Dwinelle, who, in an elaborate article, published his invention in the AMERICAN JOURNAL OF DENTAL SCIENCE, vol. v., page 278, April, 1855. I quote as follows: "Another method is to line a tooth, then solder a band of gold around it, so that it will correspond to the presenting outline of the root to be covered. * * * A gentleman of my acquaintance has, for several months past, worn an artificial cusp and crown of this general character upon a root which retains a living and healthy nerve. By accident the natural crown was broken off, but in such a manner as to leave a large portion of the central part of it covering the nerve, which protruded so far that, by cutting a groove around its base, it somewhat resembled an inverted cone; gold was packed around this until it nearly reached the outline of the root, when the prepared cusp and crown, which had been previously fitted, was secured to its place. The gold-bound cavity was then filled with gold as described."

This article is illustrated by cuts, showing the several steps to be observed in the setting of these crowns.

In an article by Dr. W. N. Morrison, of St. Louis, in the *Missouri Dental Journal*, 1869, page 184, he gives a description of a new method of setting gold crowns, of which the following is an abstract. An impression of the remaining portion of the root is obtained, a metallic die representing the form of the crown constructed; over this a thin piece of gold is swaged, accurately fitted to the root and extending below the free margin of the gum, and after soldering a bar across the inside of this crown it is fastened to the root with cement.

Dr. Beers, of California, patented a method in 1873, which is described as follows: "Fit a stout gold band around the neck of a tooth; then strike up in a piece of lead a gold crown, the size required, and run gold solder into it, to make it strong; adjust and solder it on the gold band that fits the root. Screws with T-heads are then secured in the root-canal and the crown pressed in position with hard cement."

In vol. xxii. of the *Dental Cosmos*, page 463, 1880, Dr. Talbot, of Chicago, describes an inprovement upon the Beers method by fitting a plate inside of the ring, penetrating this opposite the root canal and screwing it on the root, placing a layer of gutta-percha between root and cup. The gold crown is fitted either over or inside of this cup and also fastened with gutta-percha.

In the American Journal of Dental Science, vol. iii., page 497, 1853, Dr. A. T. Willard describes a method of pivoting. The root is cut level and below the gum, and prepared with an instrument which he calls a counter-drill. A smaller sized one is used next to cut into the inside of the root about one-sixteenth of an inch deep. The crown is prepared to fit the counter-hole in the root accurately, and has a wood pivot which is fastened in the root with mastic.

In the American Journal of Dental Science, vol. vi., page 24, 1856, Dr. John Coghlan, of Ireland, describes a capillary tube used in pivoting teeth. It was formerly applied instead of a solid gold pivot to facilitate the escape of gases and fluids from the root.

A method of pivoting teeth by Dr. T. J. Thomas is as follows: A square pivot of platinum-and-gold alloy is made by soldering two narrow pieces together at one end. The pivot is finished smooth and made accurately square. Thin platinum is bent around it, jointed and soldered. The root is prepared with under-cuts and drilled out largely in excess of the size of the pivot used. The square cylinder is fastened in the root with gold or amalgam, which, when hard, is filed smooth; a plate of soft gold is fitted to the margin of the root, and the pivot, after being placed in position is soldered to it. A plate-tooth is backed and fitted to the plate; both parts invested and soldered. The free ends of the pivot are bent apart before inserting.

In the *Dental Cosmos*, vol. ii., page 417, 1861, Dr. J. D. White describes his method: The root is enlarged for the reception of the pivot. A piece of hickory is perforated with a drill, and a gold wire forced into it; the wood condensed with a burnisher to fit the root. A natural or a porcelain crown is fitted to the root; the proper position obtained with a temporary pivot. The crown and pivot are inserted by setting a peg of wood against the cutting-edge and driving it home with a mallet without too much force. Plate-teeth are used in the following manner: "Solder a strong plate to the tooth as a backing, and to this a round wire for a pivot; drill a piece of wood to fit the gold pivot; then dress this down so as to fit the hole in the root; place it in the root, keeping it dry; dress it even with the root, and place your tooth in, and the operation is complete." In cases where the root is much decayed, it is drilled out a gold cylinder is screwed into it, and the groove between the margin of the root and the tube plugged with gold. The crown is subsequently set into this with a solid gold wire.

In vol. iii., page 614, 1862, *Dental Cosmos*, Dr. S. Richardson describes a method of using vulcanized rubber in pivoting teeth. An ordinary pivot crown is fitted to the root, leaving a space posteriorly between crown and root; the crown is attached to it with a temporary wood pivot, which is withdrawn, and soft wax interposed between the root and crown; this is pressed into the root to obtain an impression; the crown and pivot are again withdrawn; invested in plaster in such a manner as to be able to withdraw the wood pivot and remove the wax from the investment; soft rubber is now packed into the space, and a gold wire inserted to strengthen the pivot. It is then inclosed in a flask and vulcanized. Before inserting, gold foil is placed between the root and crown to make the joint perfect. By another method spring gold wire is incased in tin foil; a layer of rubber wrapped around it and vulcanized. The root must be prepared to receive the above pivot with its lining, which is forced into it. The fitting of the crown is the same as in the former method, except that no temporary pivot is used. The crown is fastened to the wire in the root with hard wax. It is withdrawn and manipulated the same as described before, except that the gold pivot is permanent, not being incased in rubber in the investment. To secure this tooth in the root the pivot is either split or flexed upon itself and then forced into the root. A plate-tooth may also be used with the above method. The root is prepared in the same manner as before; a plate-tooth backed with gold, waxed to the gold pivot, and carefully withdrawn; invested and soldered; then re-applied; soft wax pressed posteriorly upon the base of the root; this then removed and imbedded in plaster; rubber is packed into the space after removal of the wax, and the operation completed as in the former method.

In vol. v., page 218, 1863, *Dental Cosmos*, Dr. J. H. McQuillen describes the use of osteo-dentine in the setting of pivot teeth: In cases where the walls of the root are too thin to support a pivot, the root is cleansed from decay; filled nearly even to the top with osteo-dentine; a pivot tooth with wood pivot is pressed into the root. By this process the parts are firmly united.

In vol. vii., page 301, 1865, *Dental Cosmos*, Dr. C. E. Latimer, describes a method of pivoting. The nerve-canal of the root is enlarged and compactly filled with gold; a hole is drilled into the center, a little smaller in diameter than the pivot intended to be used; with a screw-tap the hole is prepared for the pivot, which consists of silver wire with a screw cut upon it; this is screwed into the root, projecting a little from the surface, around this and over the base of the root Wood's alloy is carefully worked, a rubber tooth is fitted to the space, the metal moulded around the pins and finished to imitate the shape of the natural crown.

In vol. vii., page 361, 1866, *Dental Cosmos*, Dr. I. J. Wetherbee gives the following method of applying a common pivot-tooth: A screw is cut upon a gold pivot about one-third its length and firmly inserted in compressed hickory; the end which enters the crown is provided with a dovetailed slot, which is filled with wood so as to retain the crown in position. The pivot is then carried to its position in the root, and the space between the pivot and cavity filled with gold. A cylinder of wood is inserted in the crown, placed in position and forced in the root. By another method the porcelain tooth is made with a groove in the center of its posterior surface, with two platinum pins on either side, for the purpose of receiving a gold backing and cylinder, to be soldered and finished to represent the ordinary form of pivot-teeth. The cylinder may be round or square. The insertion of the pivot is the same as in the former process.

In vol. x., page 530, 1868, *Dental Cosmos*, Dr. M. L. Battle describes his method of mounting pivot-teeth. The root is prepared with under-cuts; a gold pivot fastened into it with gold; the tooth ground to fit the root, and left about one-twentieth of an inch shorter than the tooth next to it. Amalgam is packed around the pivot and over the surface of a root. The crown is then forced on until it has the proper length. The same may be done with a wood pivot.

In vol. xi., page 1, 1869, *Dental Cosmos*, Dr. E. Blake gives his method. A thin gold tube with a longitudinal slit is forced into the root, which has been prepared for this purpose. An ordinary pivot-tooth is used by adjusting a gold or platinum pivot in the pivot-hole and flowing in solder. A way which he considers better is to cap the end of the root with thin gold plate to be retained in place by a pivot attached to it. A plain plate-tooth is backed and soldered to this plate. Another plan is the adaption of a hollow screw to the hole in the root; this is screwed into the root and the crown is attached as described before. When a root does not stand in its proper position, prepare the root with a tube: fit a pivot of gold; cover the base of the root as before, only extending the gold over the gum to the place where the new tooth is required; and mount a plate-tooth on this base.

In vol. xiv., page 583, 1872, *Dental Cosmos*, Dr. M. H. Webb describes and illustrates a method of pivoting. A heavy narrrow gold backing is attached to the crown, extending into the root, which is filled in with gold, extending over the margin of the root around a dovetail backing and built up to complete the contour on the plated portion.

In vol. xv., page 110, 1873, *Dental Cosmos*, Dr. Manuel Trujillo describes his plan of pivoting. Take an impression of the root; make die and counter-die; swage a platinum plate and trim it according to the shape of the root. Perforate the plate opposite the root-canal; make a platinum pivot and fasten through it by solder; fit a plain plate-tooth thereon and fill the palatal portion with solder. The root-canals is provided with under-cuts and the tooth fastened with bone filling.

It vol. xv., page 155, 1873, *Dental Cosmos*, Dr. E. L. Hunter describes this method: A pin of platinum alloy, with a thread cut on one end and a cleft on the other is screwed into the root, the cleft being intended to occupy the hole in the crown. Several of Mack's screws are attached to the base of the root; gold is filled around these and over the margin of the root, and the tooth is fitted to this surface and fastened by driving it over the cleft end of the pivot.

In vol. xv., page 289, 1873, *Dental Cosmos*, Dr. M. H. Webb describes gold crowns with porcelain faces. A plate-tooth with straight pins is backed with gold, the latter bent to form a cannula. A platinum-gold pivot is fitted to this, and left long enough to extend into the root. The pivot is held in the root by packing with gold, and the gold built over the margin of the root and palatal portion of the crown as well as the lateral grooves of the cannula and dovetailed cuts in the posterior surface of the porcelain face.

In vol. xv., page 503, 1873, *Dental Cosmos*, Dr. G. P. Carman describes the following plan. An ordinary pivot-tooth with a hole drilled clear through it is fitted to the root; a gold pivot is fastened in the root and split at the opposite end; the crown is set in position and the split end of the pivot filled with gold, thus holding the crown in place.

In vol. xv., page 657, 1873, *Dental Cosmos*, Dr. B. O. Doyle, gives the following method. A pivot-crown is used in this process; a cylinder of heavy gold is prepared and forced into the hole, and a metallic pivot is fastened therein with shellac. The cavity in the root is filled with powdered shellac, and the tooth and pivot are then warmed and pressed into position.

In vol. xv., page 666, 1873, *Dental Cosmos*, Dr. T. S. Phillips, of Buffalo, describes his method of pivoting with gold pivots. A gold tube with screw-cuts upon its outer side is fastened in the root; under-cuts are made for the anchorage of gold, which is built around the tube and over the margin of the root. A gold plate is fitted to this surface; A pivot smaller than the tube is soldered to this and a plate-tooth backed and soldered to the plate. Another tube is now inserted into the former, the pivot fitting this tube accurately, and the tooth is forced in position.

In vol. xvi., page 358, 1874, *Dental Cosmos*, Dr. E. W. Foster, of Boston, describes and illustrates his method. Porcelain crowns similar to the ordinary wood-pivot crown are used for this process; the hole runs through the crown and is enlarged at the posterior part, forming an open cavity. These crowns are, after being fitted to the root, screwed thereon with steel screws. Gold foil is used for a packing between the root and crown. Where the end of the root is extensively decayed, it is filled with gold. This process can be applied as well on the front as on the back teeth.

In vol. xvii., page 604, 1875, *Dental Cosmos*, Dr. T. F. Chupein, of South Carolina, communicates the following plan. The root-canal is enlarged, having a reserved coneshaped under-cut at the lower end. A gold cylinder provided with a screw at the upper end is screwed into the root and filled around with gold. A gold plate is fitted to cover the margin of the root air-tight. The pivot is of the split type; it penetrates the plate and is soldered to it. A plain plate-tooth is backed and soldered to the plate. Before inserting, the slit ends of the pivot are separated, and thus the crown is held in position by spring-power.

In vol. xviii., page 53, 1876, *Dental Cosmos*, Dr. Dwight M. Clapp, of Boston gives his plan of pivoting. The root-canal is enlarged and provided with under-cuts at the lower end. The hole is filled up, solid with gold, which extends over the face of the root. A hole is drilled in the center of the gold filling, for the reception of the pivot. The crown is fitted to the gold surface and attached with a wood pivot.

In vol. xviii., page 110, 1876, *Dental Cosmos*, Dr. J. B. Wilcox, of Michigan, proposes the following. The root is enlarged, with under-cuts; filled with amalgam, which is extended over the face of the root; an ordinary wood-pivot tooth is attached by a hickory pivot.

In vol. xix., page 481, 1877, *Dental Cosmos*, Dr. W. G. A. Bonwill's method is described and illustrated. The root is prepared with under cuts and enlarged greatly in excess of the size of the pivot that is to occupy it; and to prevent exposure of dentine, the margin is counter-sunk. The metal pivot used has an enlargement at one end and a screw-thread at the other. The enlarged part of the pin is retained in the root by amalgam, after the setting of which a full porcelain crown, with a hole running through and counter sunk at both ends is fitted; the counter-sunk portion of the crown is filled with amalgam, forced into position and screwed down with a nut. Another mode is to set a three-cornered platinum pin in the root and press the crown over it, using amalgam as before. No screws or nuts are used in this latter process.

In vol. xxi., page 232, 1879, *Dental Cosmos*, Dr. H. K. Leech describes and illustrates his plan. The root is drilled out and enlarged toward the apex. A gold tube corresponding with the drill-hole is prepared ; a plate is fitted to the face of the root, and the tube run through it and soldered. A plate-tooth is backed and fitted to the plate. The tube is now split in two or more places ; gutta-percha is laid between the root and the tooth ; the latter is warmed and pressed into position. The gold tube is filled with gold —the expansion of the split end of the tube wedging it firmly into the cavity.

In vol. xxi., page 289, 1879, *Dental Cosmos*, Dr. G. W. Weld gives a method which he terms "engrafting." The root is extracted and cut squarely off at the neck. A porcelain crown with a tapering screw baked in it is screwed into the root. Root with crown is replanted.

In vol. xxi., page 322, 1879, *Dental Cosmos*, Dr. William Jarvie, Jr., tells how to attach crowns to bicuspid and molar roots. Obtain a model, drill holes in it to correspond to the nerve-canals in the roots ; fit into these iridium wires ; make a cap for the end of the root ; solder the wires to this ; back a plate-tooth ; place it in position on the model, then invest and solder. The attachment is with gutta-percha.

In vol. xxiv., page 81, 1882, *Dental Cosmos*, Dr. Henry Weston describes his method. The pulp-canal is prepared with under-cuts and sufficiently enlarged to allow space for packing of gold, amalgam, or cement about the pin ; the latter is of platinum and iridium, spear-shaped, and notched to give firmness to its anchorage. A specially prepared crown is used for this process, concave at its lingual or palatal portion. After fitting the crown to the root and ascertaining the position of the pin, the latter is soldered to the crown. It is fastened to the root by gold, amalgam, or cement, which is packed around the pin and built over the palatal or lingual portion to the full contour of a natural tooth.

I have for several years taken a particular interest in setting artificial crowns on natural roots. It always appeared to me that it would be an inestimable benefit to the profession to possess a method of attaching artificial crowns which would combine strength, firmness, and durability, and preserve the exposed end of the root from further decay. Great efforts, as the literature shows, have of late years been made to solve this problem, yet you will agree with me that the percentage of really successful operations by the various methods heretofore enumerated is too small to be pronounced satisfactory. There is no reason why the attachment of an artificial crown to a root cannot be made as perfect as a gold filling in an accessible cavity; but the accomplishment of this with cement or other plastic filling-material, is extremely doubtful. It has been a settled ques-in my mind, for a long time, that nothing could lead to success but a method which was in accordance with true mechanical principles.

### Improved Method of Attachment of Crowns.

Fig. 1.

In my method of setting artificial crowns, I claim simplicity of construction, firmness, durability, and arrest of decay of the root (Fig.) From the follow-lowing description of my method it would seem that a failure would be almost impossible. To protect the end of the root from decay and to obtain a strong hold for an artificial porcelain crown, a gold band, properly applied, must be of the greatest benefit. I am aware of the fact that gold bands have been applied, but I am convinced that their adaptation after any of the old methods is defective. What I claim in my method is the preparation of the neck of the root by a set of instruments especially constructed for that purpose. These instruments enable the operator to obtain as nearly perfect adaptation between the gold band and the root of the tooth as can possibly be made. With reference to the upper centrals, laterals, and canines, as well as the corresponding lower teeth and bicuspids, there can be nothing more favorable than the application of this method. It is true that this process cannot be applied with the same advantage to the upper bicuspids or any of the molars, but I hope in time, if I am in any way supported by the profession, to solve that problem. I believe I will succeed in constructing a set of instruments which will prepare a root› the pulp of which is alive and may if healthy be readily kept so. But as this set of instruments is not quite completed, I will abstain from its description, and only allude to the setting of crowns which require the devitilization of the pulp, unless this organ has previously died. With these instruments a circular shoulder is turned on the neck of the root.

The alteration of the neck of the root, from an irregular cone to a cylindrical form, enables us to adapt a corresponding ring or cap. Such a cap, when fitting around as well as upon the end of the root prepared by these instuments,

FIG. 2. FIG. 3. FIG. 4.

forms an air-tight joint and consequently protects it from decay, at the same time giving the porcelain crown, when attached, a firmness which heretofore has never been obtained. The set of instruments by which the neck of the root is prepared consists of drills, reamers, and trephines. The drills (FIG. 2) are used to enlarge the root-canal for the guidance of the reamer or facing instrument and trephine. The reamers (FIG. 3) cut the surface of the root down as far as necessary. They produce a perfectly level surface and have a center-pin, which corresponds with the hole made by the drill in the center of the root, and acts as a guide. The trephine (FIG. 4) has also a center-pin, and is used to make the root cylindrical below the free margin of the gum. A set of these instruments includes different sizes of drills, with reamers and trephines corresponding in size adapted to various diameters of roots.

FIG. 5.

FIG. 6.

The ferrules or caps (FIG. 5) to fit roots which have been prepared by the above instruments are of gold, made by steel dies. They correspond exactly with the trephine in diameter and depth with allowance for sufficient expansion of the gold when forced on to the shoulder of the root, whereby a most perfect joint between cap and root is obtained. They have a stout central pivot which fits the hole in the root and gives increased strength and firmness.

The pulp-canal is enlarged with one of the drills selected with reference to the size of the root. A reamer corresponding in size is used with the dental engine to cut the root down to a perfect level. The trephine is applied in the same manner to give a cylindrical form to it, thus completing the shoulder (FIG. 6).

A steel wire corresponding in diameter with the drill which has been employed is now introduced into the root, projecting out about half an inch. It serves to indicate the exact direction of the root-canal. An impression-cup is selected with an opening opposite the missing tooth to take an impression of the root and adjoining parts. The object of the opening is to give free transmission to the wire in the root-canal. The wire protruding through the cup and impression-material is drawn out carefully before the removal of the impression-cup, which is then removed and the wire placed in its proper position in the impression. A set of brass root-models (FIG 7) corresponding in size with the instruments accompany them; one of these bearing the same number as the instrument with which the root has been prepared, is now placed on the wire in the impression, and serves to represent the prepared end of the root on the model. The impression is now ready to be filled with plaster. After the cast is obtained, we find the root-model imbedded in the plaster and the wire in its center-hole. The wire is now removed and the plaster cut from

FIG. 7.

around the root-model to the depth of the gold cap, which is ready to be placed upon it. A plain porcelain tooth (FIG. 8), as used in plate-work, is ground hollow on the inner surface to cover the outer front wall of the cap, thus hiding the gold Thin platinum backing is now adapted to the tooth, which is then ready to be placed in position on the model over the gold cap, and fastened thereon with hard wax. The united parts are removed carefully from the model, invested in sand and plaster and soldered. After polishing, the cap is ready to be forced upon the root by placing a piece of wood on the cutting-edge of the tooth and driving it home with a mallet.—*Dental Cosmos.*

## Figures and Tables

**Fig. 1. f1:**
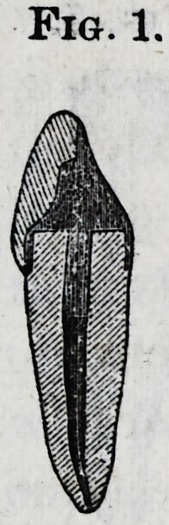


**Fig. 2. f2:**
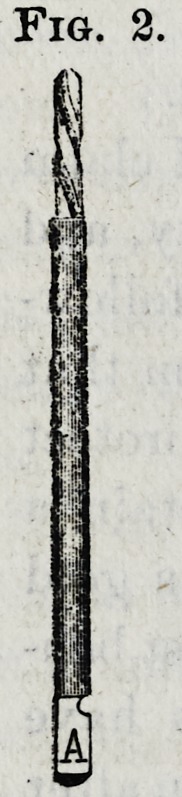


**Fig. 3. f3:**
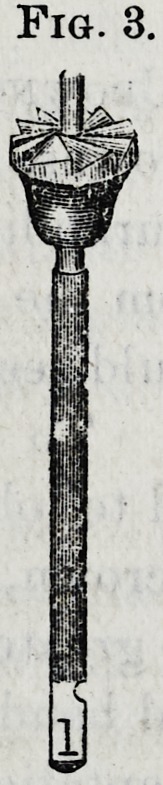


**Fig. 4. f4:**
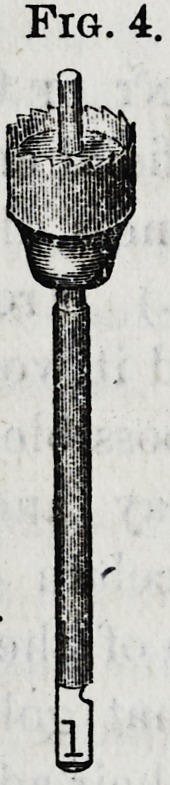


**Fig. 5. f5:**
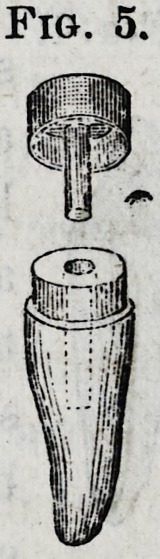


**Fig. 6. f6:**
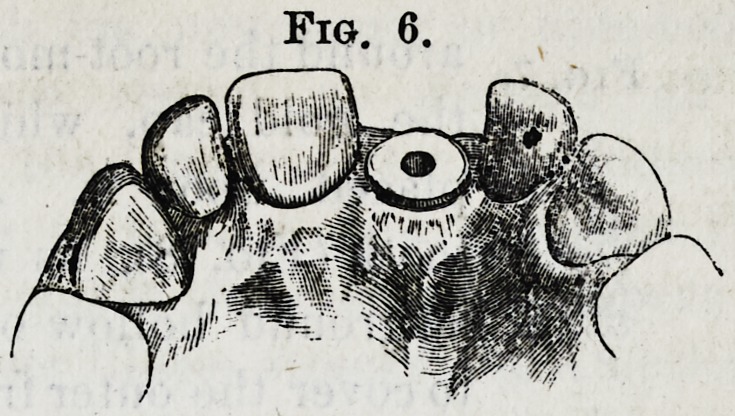


**Fig. 7. f7:**
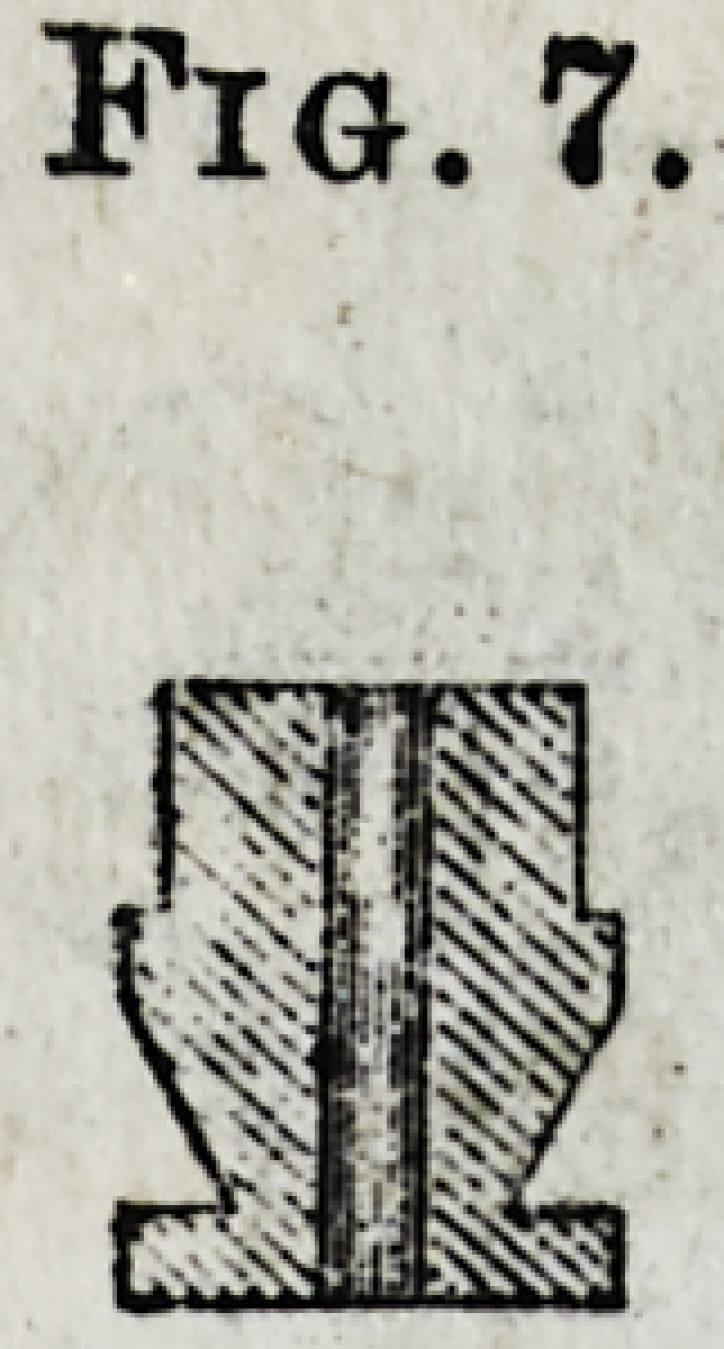


**Fig. 8. f8:**